# Effect of Peptides from Alaska Pollock on Intestinal Mucosal Immunity Function and Purification of Active Fragments

**DOI:** 10.3390/nu11102517

**Published:** 2019-10-18

**Authors:** Qiqi Li, Shikai Wang, Supanooch Poungchawanwong, Hu Hou

**Affiliations:** 1 College of Food Science and Engineering, Ocean University of China, No.5, Yushan Road, Qingdao 266003, China; 2 Laboratory for Marine Drugs and Bioproducts, Qingdao National Laboratory for Marine Science and Technology, Qingdao 266237, China

**Keywords:** Alaska Pollock, peptide, intestinal mucosal immune, SIgA, purification

## Abstract

The intestinal mucosal barrier plays an important role in systemic immune functions. This study aimed to find the mechanism of peptide from Alaska Pollock (APP) on intestinal mucosal immunity in mice induced by cyclophosphamide (Cy). Cy-induced decreases of body weight and index of immune organ were significantly improved by APP as compared with Cy group (*p* < 0.05). APP could promote the secretion of SIgA and IgA on intestinal mucosa (*p* < 0.05) and mainly had an impact on the final differentiation of IgA^+^ B cell, thereby promoting the secretion of plasma cells, which can be corroborated by the increases of IL-6 and IL-10 (*p* < 0.05). APP with high immune activity was separated and two peptides were purified and identified as Gly–Val–Ile–Lys and Ala–Cys–Asn–Gly–Arg. Therefore, APP can be considered as beneficial ingredients to protect the intestinal barrier disruption induced by Cy.

## 1. Introduction

Intestinal mucosal immunity, which is mainly composed of lymphocytes, macrophages, and plasma cells, is a natural barrier that provides the body’s first line of defense against potential damage from the environment [1]. It is widely believed that the impaired intestinal barrier, following chemotherapy drugs, is responsible for the intestinal and systemic inflammatory responses and caused diarrhea, enteritis, and even systemic immunodeficiency which can lead to high mortality [2]. Therefore, the protection of intestinal mucosal immune function is considered very important for body healthy [3].

When the small intestinal mucosa is exposed to antigen, the lymphoid tissue in the mucosa immediately produce an immune response and secrete immunoglobulin to prevent bacteria, viruses and other harmful antigens from invading the small intestine [4]. Secretory immunoglobulin A (SIgA) is the major immunoglobulin of the small intestinal mucosa. SIgA is formed by the combination of pIgR, J-chain and α-chain (IgA) and is mainly secreted by mature plasma cells in small intestinal mucosa. The secretion of SIgA on small intestinal mucosa plays a leading role in maintaining intestinal homeostasis and the balance of immune system [5]. 

It is well known that peptide obtained from marine fish could improve the body’s immunity [6]. Administration of peptides from Alaska Pollock in early basic enteral nutrition (EN) intervention had a positive effect on immunomodulatory functions. Peptide from Alaska Pollock can enhance the body’s immunity and improve the burn-induced intestinal barrier disruption and intestinal tight junction integrity [3,7]. However, the relationship between the effect on the small intestinal mucosal immunity and body’s immunity is not clear. There were few reports about peptide focused on improvement of the secretion of SIgA on the small intestinal mucosa to increase the immunity. Thus, oral administration of peptide to promote the secretion of SIgA on small intestinal mucosa for improving the immunity need to be further studied. 

Recently, lots of immunomodulatory peptides have been separated from various food resources including Chum Salmon, whey, rice protein, soybean protein, and sheep bone protein [8,9,10,11,12,13]. However, the center of the active fragment is not clear especially on the intestinal mucosal immunity.

Therefore, the study was investigated the effect of peptide on intestinal mucosal immune function by the secretion of SIgA. The spleen index and thymus index, histological assessment of the intestinal mucosa were used for evaluating the intestinal mucosal immune function in Cy-induced model. And the mechanism of peptide from Alaska Pollock on SIgA secretion was clarified through the component of SIgA and the factors of differentiation of IgA^+^ B cells. Furthermore, the sequence of peptides with high immune functions were separated and identified.

## 2. Materials and Methods 

### 2.1. Materials

Alaska Pollock was purchased from Qingdao Fusheng Food Co., Ltd. (Qingdao, China), and stored at −20 °C. Papain and trypsin were purchased from Nanning Pangbo Biological Engineering Co., Ltd. (Nanning, China). Male Balb/c mice (7–8 weeks old, body weight: 18 to 22 g) and standard rodent chow were purchased from Pengyue Experimental Animal Breeding Co., Ltd. (Jinan, China) and the permit number of mice was SCXK (Lu) 20140007. Cyclophosphamide (Cy) was obtained from Jiangsu Shengdi Pharmaceutical Co., Ltd. (Jiangsu, China). Thymosin enteric-coated tablets was obtained from Xi’an Disai Biological Medicine Co., Ltd. (Xi’an, China). Elisa assay kits of SIgA, IL-6, IL-10, IgA, and IgG was obtained from Nanjing Jiancheng Institute of Bioengineering (Nanjing, China). Other reagents were of analytical grade.

### 2.2. Preparation of Alaska Pollock Peptide (APP)

Alaska Pollock protein was hydrolyzed by 1% (*w*/*w*) papain and 1% (*w*/*w*) trypsin at a substrate-to-water of 1:2 (*w*/*v*) under constant agitation at 50 °C for 3 h with pH of 7.5. The supernatant was collected after centrifugation, and Alaska Pollock peptide (APP) was obtained after lyophilization.

### 2.3. Amino Acid Composition of APP

APP was hydrolyzed with HCl (6 mol/L) in the ampoules which were sealed with nitrogen and maintain 24 h at 110 °C, and Hitachi 835-50 amino acid analyzer (Hitachi, Tokyo, Japan) was used to analyze the content of amino acids [14].

### 2.4. Identification of Fingerprint Mapping of APP

Fingerprint mapping was identified by using Agilent G6540 Q-TOF high-resolution mass spectrometer that equipped with the electrospray ionization (ESI) interface (Agilent Corporation, Santa Clara, CA, USA). The mass spectrometer was set from 400 to 2000 m/z on the MS [15].

### 2.5. Animal Experiments

The healthy male Balb/c mice were maintained in an alternating room for 12 h light-dark cycle with a humidity of 50%–60% and temperature of 24 ± 1 °C [16,17]. After adaptive feeding of experimental mice for one week, mice (*n* = 60) were divided into 6 group (*n* = 10 for each group), including a normal control (given saline, NC), a model group (injected with Cy, given saline, Cy), a APP low-dose group (injected with Cy, given 50 mg/kg body weight/day of APP, APP-L), a APP medium-dose group (injected with Cy, given 200 mg/kg body weight/day of APP, APP-M), a APP high-dose group (injected with Cy, given 500 mg/kg body weight/day of APP, APP-H) and a positive control group (injected with Cy, given with 50 mg/kg body weight/day of thymosin enteric-coated tablets, PC). 

All of the mice had free access to standard rodent chow and cleaning water, and were given test substance once a day for 27 days. All groups except NC group were intraperitoneally injected with Cy (50 mg/kg body weight/day), and the NC group was injected with an equal volume of sterile saline on the 25th and 26th day. All mice were fasted for 24 h and blood was taken on the 27th day. At the end of the experiments, mice were euthanized by cervical dislocation.

This study was approved by the ethical committee of experimental animal care at College of Food Science and Engineering in Ocean University of China (Qingdao, China). All animal experiments were performed follow the guidelines of National Institutes of Health guide for the care and use of Laboratory animals (NIH Publications No. 8023, revised 1978).

### 2.6. Thymus Index and Spleen Index of Mice

The body weight of the mice was weighed every other day during the breeding period. Balb/c mice were euthanized after fasted (keep the supply of drinking water) for 24 h, and then the thymus and spleen were excised separately to weight. The filter paper were used to absorb the surface liquid.

Thymus index = (thymus mass (mg)/body weight (g)) × 10(1)

Spleen index = (spleen mass (mg)/body weight (g)) × 10(2)

### 2.7. H&E Staining

The segments of intestinal jejunum (1–2 cm length) were fixed in 4% (*v*/*v*) neutral formaldehyde for 12 h. Serial sections (7 μm) were stained with eosin and hematoxylin to assess the structural integrity of the intestinal mucosa. An Olympus DP70 digital camera system (Olympus Optical Co., Ltd., Tokyo, Japan) was used to record the images at 40× magnification.

### 2.8. Elisa Analysis

Jejunum (5 cm length) was obtained from mice and then were weighed. Immediately small intestinal mucus was collected and was completely into PBS buffer (containing 0.01 mmol/L PMSF, pH 7.4). After centrifuged at 4 °C, the supernatant was collected and assayed for the contents of SIgA, IgA, IgG, IL-6, and IL-10 using ELISA assay kits. IgA, IgG in serum were assayed using the same method. The specific experimental procedure was carried out according to the ELISA assay kit.

### 2.9. Quantification of mRNA Level Using RT-qPCR

The RNA was extracted from supernatant of the small intestine homogenate using Trizol^®^ reagent (Thermo Fisher Scientific, Waltham, MA, USA). Each sample (approximate 100 mg) was homogenized in Trizol^®^ reagent, and then chloroform (0.2 mL) was added to remove the ribosome. Isopropanol was added to the supernatant and mixed thoroughly. After centrifuged at 10,000 r/min for 10 min at 4 °C, the precipitate was washed repeated by 75% ethanol for 2–3 times to remove the supernatant. The precipitate was added with an appropriate amount of DEPC to obtain a pure RNA sample. The integrity of RNA was confirmed using 1% agarose gel electrophoresis. Concentration and purity of RNA were quantified by ultra-micro spectrophotometer (Nanodrop ND-2000, Thermo Fisher Scientific, Waltham, MA, USA). Then RNA was reverse transcribed into cDNA according to the Transcriptor cDNA Reverse Transcription kit (Roche, IN, USA).

For the RT-qPCR assay, the reaction volume was 20 µL, consisting of 10 μL of TransStart^®^ Tip Green qPCR Super Mix (Roche, Basel, Switzerland), 0.8 µL of primers (0.4 µL each of upstream and downstream primers at 10 µM concentration), 2 μL cDNA and 7.2 μL ultra-pure water. Data were analyzed by the comparative Ct method (2^−∆∆Ct^) and presented by using β-actin mRNA level as internal reference [18]. The sequence of the primers used in study were listed in Table 1.

### 2.10. Purification and Identification of Peptide with High Immune Activity

Firstly, APP was isolated and purified using SP Sephadex C25 cation exchange column (2.6 cm × 45 cm). The selected fragments were further separated using Sephadex G25 gel column (1.6 cm × 80 cm) and were subjected for proliferation activity of spleen lymphocytes. Samples with high proliferation activity were separated using a reversed-phase high performance liquid chromatography system (Agilent 1100, Palo Alto, USA) on a Zorbax SB-C18 semi-preparative column (9.4 mm × 250 mm). The samples were linearly eluted using acetonitrile (5%–30% (*v*/*v*)) containing 0.1% trifluoroacetic acid (TFA) with a flow rate of 2.0 mL/min for 30 min at 35 ℃. Samples were monitored at 220 nm. The sequence analysis of peptide with high proliferation activity of spleen lymphocytes were measured by using a (Q Exactive) benchtop quadrupole-orbitrap high resolution mass spectrometer (Thermo Fisher Scientific, Waltham, MA, USA) [19].

### 2.11. Determination of Proliferation Activity of Spleen Lymphocytes

The spleens were rinsed with DPBS buffer solution containing antibiotic (1000 U/mL), which were gently triturated and filtered through a 200-mesh nylon screen [20]. The spleen cells were transferred to tubes and centrifuged at 1000 r/min for 5 min. After removed the red blood cells, the precipitation was washed repeatedly and continued to cultivate on RPMI 1640 complete medium (Thermo Fisher Scientific, Waltham, MA, USA) including 10% calf serum and 100 U/mL antibiotic in 5% CO_2_ incubator for 4 h at 37 °C.

The cell viability was checked using trypan blue staining (if the number above 95%, it can be used for experiments). Spleen lymphocyte suspension (2.0 × 10^6^/mL, 100 μL) and samples (500 μg/mL, 100 μL) were added to each well in a 96-well plate, and the cells were cultured in 5% CO_2_ incubator at 37 °C for 36 h. MTT (5 mg/mL, 20 μL) was added and the plates were incubated for another 4 h under the same conditions. After incubation, the samples were centrifuged, and the supernatant was discard. Then DMSO (200 μL) was added to measure the OD value of each well at 570 nm. 

### 2.12. Statistical Analysis

The experimental data were expressed as mean ± standard deviation, and the data were analyzed using SPSS 11.5 statistical software (SPSS Inc., Chicago, IL, USA). The significant difference was statistically significant at *p* < 0.05.

## 3. Results and Discussion

### 3.1. Amino Acid Composition of APP

The amino acid composition of APP was shown in Table 2. Gly, Glu, Ala, Lys, and Asp were rich in APP, similar to that of peptide from Pacific cod and Atlantic salmon [21,22]. Essential amino acids (Leu, Thr, Ile, Phe, Lys, Met, Val, Trp) of APP accounted for 30.8% of the total amino acids and branched-chain amino (Ile, Val, Leu) occupied 40.6% of the essential amino acids. Peptides with abundance of glutamine were frequently used to improve the immunity of the small intestine and the supplementation of branched-chain amino acid played an important role on the immune response [23,24]. According to the reference protein suggested by FAO/WHO [25], the chemistry scores of essential amino acids were more than 0.8, which indicated that APP can be used as a good source of quality protein. Thus, supplements of APP could provide nutrition support for various metabolic activities to improve the small intestinal immunity [26,27]. 

### 3.2. Characteristic Fragment of APP

The peptide sequences of APP were analyzed by using ProteinPilot software, and the target peptide sequence in the experiment was screened using the database about protein peptide from Alaska Pollock. As shown in Table 3, the molecular weight of APP was ranged from 700.32 Da to 1488.70 Da, and the fragments of Glu–Glu, Gly–Arg, and Gly–Glu were repeated in APP. Branched-chain amino acid residues exhibited in the middle of the characteristic peptide and Lys repeatedly appeared at the end of peptide sequences. The results were similar to other study, which supported that Gly–Glu and Glu–Gln are the characteristic fragment of peptide from Alaska Pollock [15]. It can be seen from the structural analysis of these immunologically active peptides, almost all peptide sequences contain hydrophobic amino acids and most of them possess hydrophobic amino acids as N-terminally. N-terminus of peptide which show the high immunological activity is a hydrophobic amino acid [28]. 

### 3.3. Changes of Thymus Index and Spleen Index in Mice

**Figure 1:**
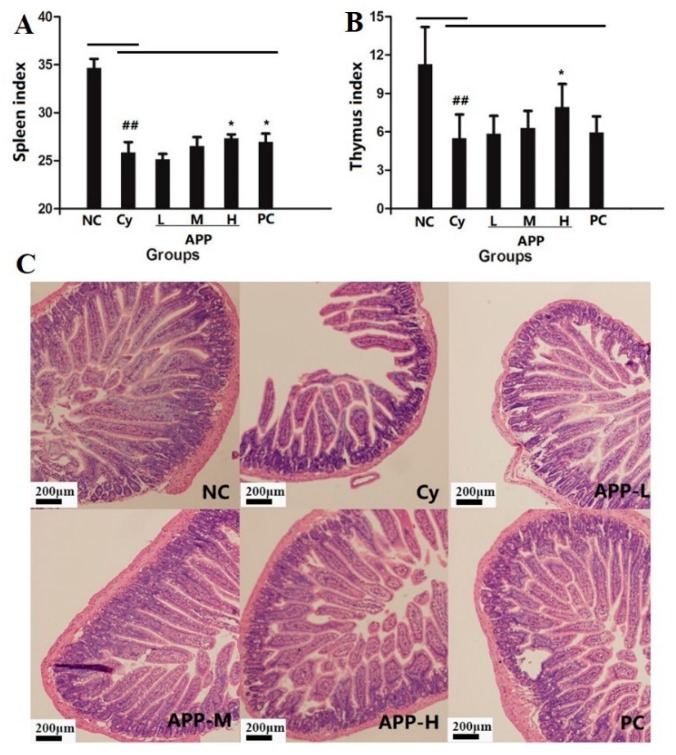
The change of spleen index (**A**), thymus index (**B**) and morphology of small intestine (**C**) in mice. Values are expressed as mean ± SD, *n* = 10. The different symbols indicate significant differences (^#^ *p* < 0.05, ^##^ *p* < 0.01 compared to the NC group; ^*^ *p* < 0.05, compared to the Cy group).

The thymus index and spleen index could reflect the capacity of immunity [29]. As shown in Figure 1A,B, the spleen index and thymus index of the Cy group were significantly lower than those in NC group (*p* < 0.01), which suggested that Cy had an effect on the weight reduction of immune organs. The APP-H group can improve the spleen index and thymus index in immunocompromised mice induced by Cy (*p* < 0.05); however they were not up to the normal level, which indicated that APP could improve the immune organ index of mice with low immunity but cannot reverse the atrophy of thymus and spleen. The results were consistent with the previous study [8]. It was reported that many bioactive substances can enhance the immunity by increasing the organ index, such as pineal peptide, collagen peptide from jelly fish, and squid ink polysaccharide [30,31,32].

### 3.4. Histological Assessment of the Intestinal Mucosa

Cy could cause low immunity, while the intestinal tract as the body’s immune organ was easily affected [33]. As shown in Figure 1C, the results showed the small intestine of the NC group was intact, the villi were slender, the arrangement was tight and orderly, and the brush edge was clear. After 2 days of intraperitoneal injection with Cy (50 mg/kg), the villi in Cy group were characterized by marked atrophy, thick, broken, and arranged loosely as well as loss of partial epithelial cells [34]. The phenomenon indicated that Cy had caused great damage to intestinal histomorphology, indicating that the model was successful. In APP group, the length of intestinal villi increased and the histomorphology recovered, suggesting that APP could have a certain degree of improvement in the morphology of small intestine. Similarly, collagen peptides from Alaska Pollock skin were reported for improvement of intestinal barrier disruption in burn-induced mice [35].

### 3.5. Effect of APP on the Secretion of SIgA in Small Intestinal Mucosa

**Figure 2:**
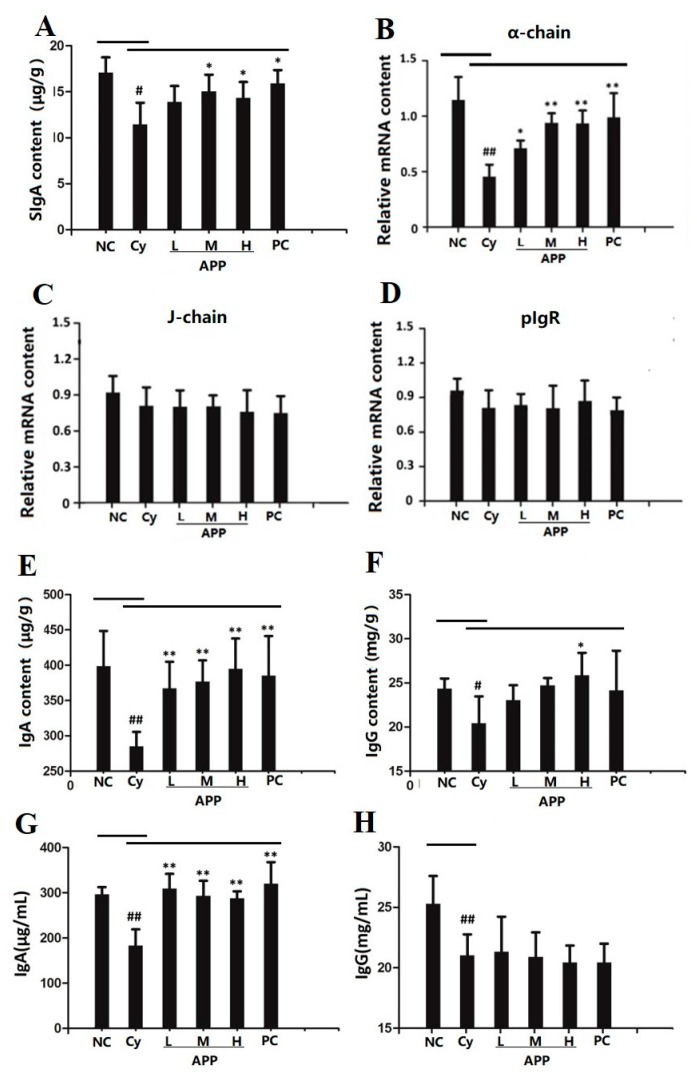
The secretion of secretory immunoglobulin A (SIgA) (**A**), mRNA levels of α-chain (**B**), J-chain (**C**) and pIgR (**D**), content of IgA (**E**), IgG (**F**) in intestinal mucosal and IgA (**G**), IgG (**H**) in serum. Values are expressed as mean ± SD, *n* = 10. The different symbols indicate significant differences (^#^ *p* < 0.05, ^##^ *p* < 0.01 compared to the NC group; ^*^ *p* < 0.05, ^**^ *p* < 0.01 compared to the Cy group).

SIgA is an immune molecule secreted into the small intestinal lumen, and damage of acquired immunity of the small intestinal mucosa are related to a reduction of SIgA levels in lumen [36]. As shown in Figure 2A, compared with the NC group, the secretion of SIgA in Cy group was significantly decreased after treated by Cy (*p* < 0.05), which suggested that Cy can significantly damage the epithelial cells, resulting in reduced secretion of SIgA. In contrast, APP-M, APP-H, and PC groups significantly improved the secretion of SIgA (*p* < 0.05) and the results were depend on dose of peptide. The results indicated that APP could increase the small intestinal mucosal immunity by promoting the secretion of SIgA in the small intestinal mucosa. Glycinin could increase the secretion of SIgA in intestinal mucosa to promote the immune function [37]. Partial enteral nutrition could increase the content of SIgA in mice in a dose-dependent way to reduce the inflammation and infection [38].

### 3.6. Effect of APP on J Chain, pIgR, and α-chain on mRNA Level

It had been supposed that APP significantly increase the secretion of SIgA in immunocompromised mice. In order to further explore the mechanism of APP to promote the secretion of SIgA, the effects of three components of SIgA (α-chain (IgA), J-chain and pIgR) were further studied. There were no significant differences in the relative level of J-chain and pIgR among different groups (in Figure 2C,D), which indicated that Cy and APP have no significant effect on J-chain and pIgR. As shown in Figure 2B, compared with Cy group, the relative level of α-chain (IgA) in APP-L group was increased (*p* < 0.05), and that in APP-M, APP-H group, and PC group was significantly improved (*p* < 0.01). The results showed that APP can significantly improve the relative level of IgA and this is also consistent with the results about the increased content of SIgA which can be formed by IgA. The similar researches had supported that significant increase of IgA secretion in the lamina propria of the small intestine played an important role on improvement of intestinal immune function [39].

### 3.7. Effect of APP on IgA and IgG in Small Intestine and Serum

IgA produced by the immune cells could convert into SIgA through the transcellular transport between intestinal epithelial cells [40]. Thus, the potential capacity of small intestinal mucosal immunity can be reflected by the secretion of IgA. As shown in Figure 2E, IgA content in Cy group was significantly lower than that in NC group (*p* < 0.01). However, the APP group and PC group significantly increased the content of IgA (*p* < 0.01). In Figure 2F, APP-H group significantly improved the amount of IgG in intestine compared to the Cy group (*p* < 0.05). The results indicated that APP can prevent the intestinal mucosal damage by promoting the secretion of IgA and IgG, and further accelerate the secretion of SIgA. Similar results have been shown in the previous studies that soy bioactive peptides could promote the level of IgA in intestine to improve the immunity [41].

It is well known that IgA is enriched in the intestinal mucosa lamina propria, and IgG is a predominant immunoglobulin in serum to play a role in body’s immunity. So IgA and IgG in serum were studied for further insight into the body’s immunity. As shown in Figure 2G,H, the levels of IgA and IgG in serum in Cy group were significantly reduced compared with the NC group (*p* < 0.01), which showed that Cy can significantly damage the mucosal immunity and body’s immunity. Mice in APP group and PC group had significantly increased content of IgA in serum (*p* < 0.01). However, they had no significant effect on IgG levels in serum, which suggested that the improvement of intestinal mucosal immunity by APP might be greater than the body’s immunity. The results were consistent with the experimental results about the study on squid ink polysaccharide [42].

### 3.8. Effects of APP on the Factors of Differentiation of IgA^+^ B Cell

#### 3.8.1. Effects of APP on the Factors of Early Differentiation of IgA^+^ B Cell

**Figure 3:**
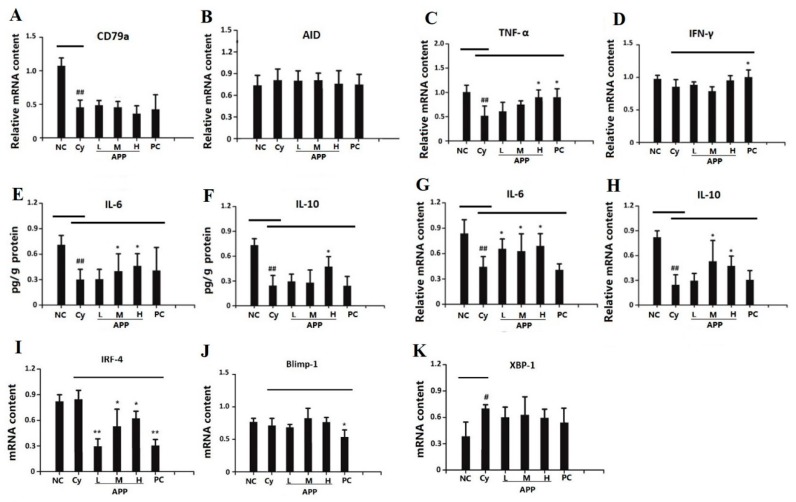
Effects of APP on the factors in the differentiation of IgA^+^ B cell and effect on plasma cell differentiation. CD79a mRNA level (**A**) and AID mRNA level (**B**) are the factors in the early differentiation of IgA+ B cell. TNF-α mRNA level (**C**) and IFN-γ mRNA level (**D**) are the factors in the medium-term differentiation of IgA+ B cell. IL-6 protein expression (**E**), IL-10 protein expression (**F**), IL-6 mRNA level (**G**) and IL-10 mRNA level (**H**) are the factors in the final differentiation of IgA+ B cell. IRF-4 (**I**), Blimp-1 (**J**), and XBP-1 (**K**) are the transcription factors in plasma cells. Values are expressed as mean ± SD, *n* = 10. The different symbols indicate significant differences (^#^ *p* < 0.05, ^##^ *p* < 0.01 compared to the NC group; ^*^ *p* < 0.05, ^**^ *p* < 0.01 compared to the cyclophosphamide (Cy) group).

The increased content of IgA depends on the promotion of plasma cells which can be differentiated by IgA^+^ B cell [40]. CD79a is a specific antigen for the early differentiation of IgA^+^ B cells. Experimental results (Figure 3A) showed Cy significantly reduced the relative mRNA level of CD79a (*p* < 0.01) compared with NC group. Cy caused damage to the early differentiation of IgA^+^ B cell, resulting low immunity in mice. There was no significant difference on the CD79a content in APP group and PC group compared with the Cy group, indicating APP have no significant improvement on the early differentiation of IgA^+^ B cells. The existing research is similar to the previous results [43]. In the stage of CSR, differentiation of B cell mainly relies on the intracellular AID enzyme to mediate the conversion of IgM^+^ IgD^+^ B cells into IgA^+^ B cells [42]. As shown in Figure 3B, the relative mRNA level of AID enzymes was not changed significantly, suggesting that there was no remarkable effect of APP on the stage of CSR. However, in the existing studies, the immunoregulatory peptide prepared from whey protein played a role in promoting secretion of the AID enzyme in the stage of CSR [44].

#### 3.8.2. Effects of APP on the Factors of Medium-Term Differentiation of IgA^+^ B Cell

In the medium-term differentiation of IgA^+^ B cells, IgM^+^ IgD^+^ can stimulate normal B cells and produce cytokines such as TNF-α, IFN-γ, and TGF-β that are closed related with immunity [45]. This experiment mainly examined the effects of APP on TNF-α and IFN-γ which can be used as markers of medium-term differentiation of IgA^+^ B cells [46]. As shown in Figure 3C, the relative content of TNF-α in Cy group was significantly decreased (*p* < 0.01) compared with NC group, and that in APP-H group and PC group increased significantly (*p* < 0.05). However, APP and Cy have no significant effect on the content of IFN-γ (shown in Figure 3D). Up-regulation of the relative mRNA level of TNF-α was a predictor of the improvement of immune cell functions, which indicated that APP could increase the secretion of TNF-α. According to the previous study, peptide could affect the content of IgA most in intestinal epithelial cells and then exerted intestinal mucosal immune regulation [47].

#### 3.8.3. Effects of APP on the Factors of Final Differentiation of IgA^+^ B Cell

Some interleukins are involved in the regulation of cell maturation. IL-6 and IL-10 are important markers in the final differentiation of IgA^+^ B cells [48]. Therefore, this experiment examined IL-6 and IL-10 on the mRNA and protein levels. As shown in Figure 3E–H, the content of IL-6 and IL-10 had significantly decreased in Cy group compared with that in NC group (*p* < 0.01), and APP can effectively improve protein expression and mRNA level of IL-6 and IL-10 (*p* < 0.05). Previous studies had confirmed that IgA^+^ B cells undergo the stage of CSR and SHM with the help of IL-6 and IL-10, and IL-6 could also increase production of immunoglobulin by 6–8 times [49]. APP could play an important role on final differentiation of IgA^+^ B cell, thereby improving intestinal mucosal immunity.

In summary, APP had an important role on final differentiation of IgA^+^ B cell, thereby promoting the secretion of plasma cells and increasing the content of IgA. Thus, APP was supported to promoting the secretion and synthesis of SIgA in intestinal mucosal immunity.

### 3.9. Effects of APP on the Differentiation of Plasma Cell

According to the experimental results above, APP mainly played an important role on the final differentiation of IgA^+^ B cells. So, it is necessary to further explore whether APP affected the differentiation of plasma cell [50]. When B cells differentiated into plasma cells, mature protein (Blimp-1) can be induced and regulated the differentiation of plasma cell [51]. XBP-1 is a differentiation factor that is essential for the stage of B lymphocytes into plasma cells [52]. IRF-4 regulated the differentiation of pre-B cells into immature SIgM^+^ B cells and can regulate the final differentiation of IgA^+^ B cells [53]. In summary, IRF-4, Blimp-1, and XBP-1 are important differentiation factors in plasma cells. As shown in Figure 3K, the relative level of XBP-1 was significantly increased in the Cy group (*p* < 0.05) compared with NC group, but APP had no significant effect on XBP-1. The relative level of Blimp-1(shown in Figure 3J) showed no significant difference among NC, Cy and APP groups. For IRF-4 level (shown in Figure 3I), there was no significant difference between Cy group and NC group. However, in another study, squid ink polysaccharide were reported to have a good effect on the content of IRF-4 to improve the stage of plasma cells differentiation [42].

### 3.10. Purification and Identification of the Peptide Fraction with High Immunological Activity

**Figure 4:**
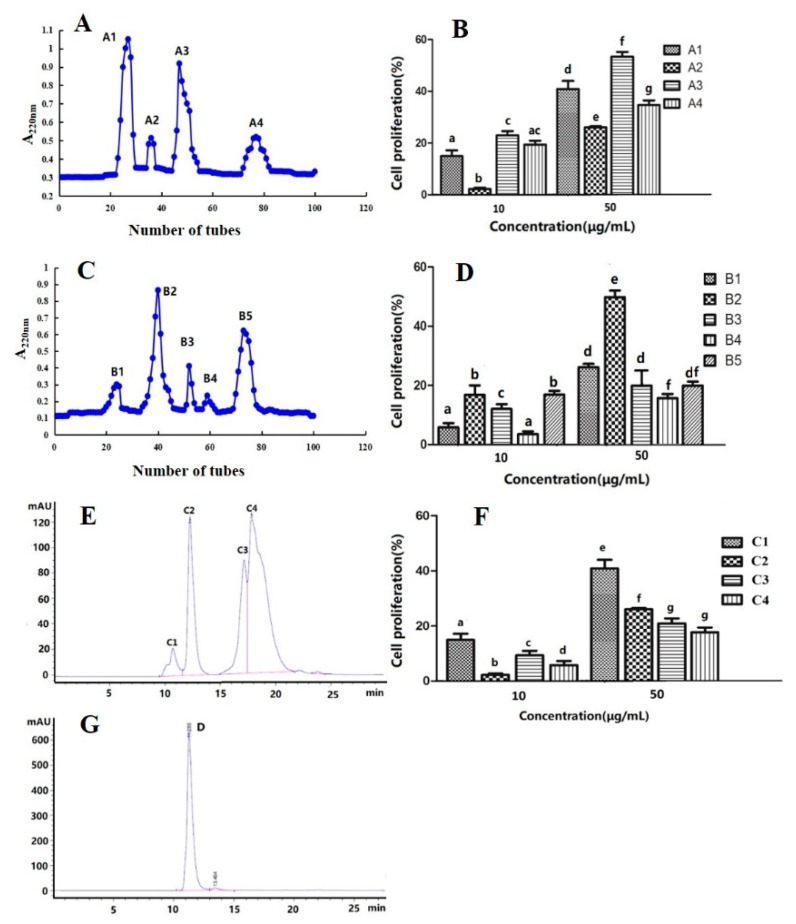
Elution curve of APP and effects of peptide fractions on spleen lymphocyte proliferation. (**A**) SP Sephadex C-25 elution curve of APP. (**B**) Effects of peptide fractions separated by SP Sephadex C25 on spleen lymphocyte proliferation. (**C**) Sephadex G25 elution curve of A3 peak. (**D**) Effects of peptide fractions of A3 peak on spleen lymphocyte proliferation. (**E**) RP-HPLC elution curve of B2 peak. (**F**) Effects of RP-HPLC elution fractions of B2 Peak on spleen lymphocyte proliferation. (**G**) RP-HPLC elution curve of C1 peak. The different letters indicate significant differences (*p* < 0.05).

Peptide through the separation and purification showed the high immune activity. The SP Sephadex C25 cation exchange column was utilized to separate the immunomodulatory peptides from Alaska Pollock frame and the highest activity was the fifth peak [7]. A novel immunomodulatory hexapeptide was purified from silkworm (Bombyx mori) pupa protein hydrolysates using sephadex gel filtration chromatography and reverse-phase high-performance liquid chromatography [54,55]. As shown in Figure 4A, the components of APP were separated by SP Sephadex C25 cation exchange column, and four peaks of A1, A2, A3, and A4 were obtained. Among all fractions, A3 peak exhibited the most potent spleen lymphocyte proliferation activity (shown in Figure 4B). At the sample concentrations of 10 μg/mL and 50 μg/mL, the spleen cell proliferation activities were 22.3% and 51.4% respectively. Therefore, A3 peak was further separated and purified using a Sephadex G25 gel column. In Figure 4C,D, five peaks of B1, B2, B3, B4 and B5 were isolated and the highest activity was B2 peak, and the spleen cell proliferation activities were 17.8% and 47.9% respectively at 10 μg/mL and 50 μg/mL. Subsequently, B2 peak was further purified by using reverse-phase high performance liquid chromatography. As shown in Figure 4E,F, C1, C2, C3 and C4 were obtained and the C1 peak have the strongest immune activity with the proliferation activities were 14.2% and 39.7% at 10 μg/mL and 50 μg/mL. Therefore, the C1 peak was further eluted by reverse-phase high performance liquid chromatography to obtain a single and symmetrical peak named D as shown in Figure 4G. 

**Figure 5:**
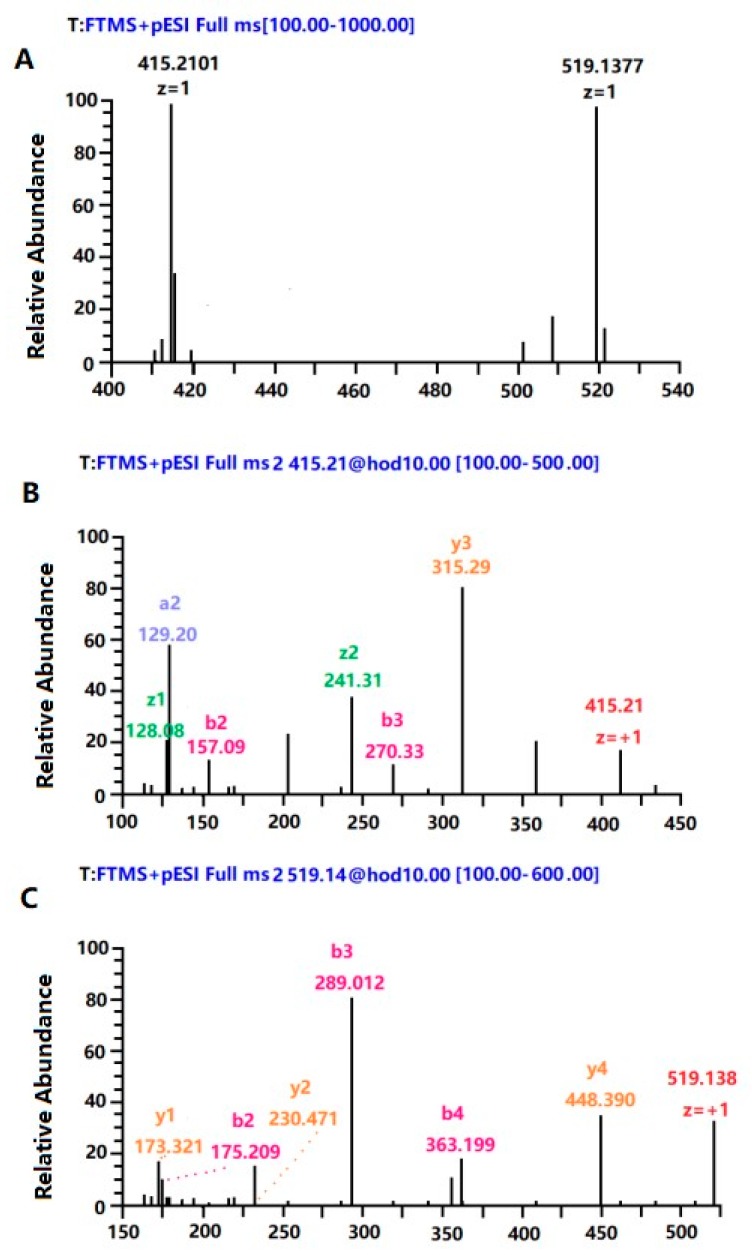
ESI-MS spectra analysis of peak D. (**A**) The MS spectrum of peak D. (**B**,**C**) The MS/MS spectrum of D peak.

It can be seen in Figure 5A, the exact molecular weights of the two peaks with the highest response values in the peak D peptide were 415.2 and 519.1 respectively. The two peaks were subjected to two-stage mass spectrometry using Nano-ESI-Ms/Ms as shown in Figure 5B,C. The composition had an exact molecular weight of 415.2 Da, which was a tetrapeptide, and its sequence was Gly–Val–Ile–Lys with the spleen lymphocyte proliferation rate of 30.12% at 20 μg/mL. The other peptide is pentapeptide, and its sequence is Ala–Cys–Asn–Gly–Arg with spleen lymphocyte proliferation rate of 29.72% at 20 μg/mL. Active peptide extracted from milk with the sequence of Thr–Thr–Met–Pro–Leu–Trp and Val–Arg–Gly–Pro–Phe–Pro–Ile–Val, which showed strong immunological activity in vitro [56,57]. Peptide with high immune activitity was isolated from rice with a sequence of Gly–Tyr–Pro–Met–Tyr–Pro–Leu–Pro–Arg [58]. The pure peptide that was identified have the high immunity maybe could promote the secretion of SIgA on the small intestinal mucosa, and improve the immunity on small intestinal mucosa.

## 4. Conclusions

Peptide obtained from Alaska Pollock can improve the small intestinal mucosal immunity by improving the immune organ index, recovering structure integrity of intestinal mucosa, increasing the secretion of SIgA, IgA, IL-6, and IL-10 that related to the intestinal mucosal immunity. Furthermore, effective fragments of APP with high immune activity were identified. 

## Figures and Tables

**Table 1:** Specific primer sequences used for RT-qPCR.

Gene	Forward Primer	Reverse Primer
β-actin	CAGGCATTGCTGACAGGATG	TGCTGATCCACATCTGCTGG
pIgR	CAGAAATAGCATGGCAGACTTCAA	TGCCGACTAGGCCATGTCAG
J chain	TTCGTTAAGGCTGTCCTTGT	AAGCGGAGCTGGAAGATCAG
α-chain	TGAGAGCTGGAACAGTGGCG	TCAGCGCCAGCTCCTCCGAC
TNF-α	CTGTACCCCACGTCGTAGC	TTGAGATGCATGCCGTTG
IFN-γ	CACTGCGTCTTGGCTTTGCA	GCTGATGGGCTGATTGTCTTTC
IL-10	ACTGCAGCCACGTCGTAGC	TGTCCACCTGGTCCTTTGTT
IL-6	GGTACCAAACTGGATATAATCAGAA	CAAGGTAGCTATGGTACTCCAGC
AID	GGGAAAGTGGCAATCACCTAT	CGTTCGGCAAAGCGTCAT
CD79a	TCAACACTGCCAGGGAACC	GGGGAAGACAGACAGCAAGAAC
Blimp-1	TTTGTGGACAGAGGCCCAGT	AAAGCGTGTTCCCTCCGGTAT
IRF-4	CGGGCAAGCAGGCCTACAAT	TGGCTCCCTCCGGAACAATC
XBP-1	GCTGCGGAGGAAACTGCAAA	TCCATTCCCACGCGTGTTCT

**Table 2:** Amino acid compositions of Alaska Pollock peptide (APP) (mol/1000 mol).

Amino Acid	RP ^a^	APP	
		Content	CS
Leu	70	67	0.96
Thr	40	40	1.00
Ile	40	26	0.65
Phe	60	34	0.57
Lys	55	74	1.35
Met	35	32	0.91
Val	50	35	0.70
Trp	10		
Pro		11	
Asp		77	
Tyr		13	
Ser		53	
Glu		119	
Ala		91	
Cys			
Arg		44	
Gly		259	
His		25	

^a^ Reference protein, essential amino acids of reference protein according to FAO/WHO (1985). CS: Chemistry scores.

**Table 3:** Main peptide sequences analysis from APP.

Peptide Sequence of APP	Molecular Mass (Da)	Peptide Sequence of APP	Molecular Mass (Da)
Ala–Gly–Asp–Asp–Ala–Pro–Arg	700.32	Leu–Asp–Phe–Glu–Asn–Glu–Met–Ala–Thr	1068.45
Ser–Leu–Ser–Asp–Leu–Asn–Pro	745.35	Gly–Thr–Glu–Asp–Glu–Leu–Asp–Lys–Tyr	1068.46
Gly–Ser–Leu–Glu–Gln–Glu–Lys	789.38	Glu–Thr–Leu–Asp–Met–Leu–Glu–Thr–Met	1087.49
Gly–Val–Glu–Glu–Asp–Leu–Met	791.34	Gln–Glu–Tyr–Asp–Glu–Ala–Gly–Pro–Ser–Ile	1090.45
Gly–Gly–Asp–Asp–Leu–Asp–Pro–Asn	801.32	Ile–Glu–Glu–Leu–Glu–Glu–Glu–Ile–Glu	1131.52
His–Leu–Asp–Asp–Ala–Val–Arg	824.42	Ser–Ser–Pro–Gly–Asp–Asp–Asp–Met–Ala–Asn–Lys	1135.45
Gly–Val–Glu–Asp–Asp–Ser–Val–Gln	847.36	His–Glu–Leu–Glu–Glu–Ala–Glu–Glu–Arg	1140.51
Ala–Leu–Thr–Asp–Ala–Glu–Thr–Lys	847.43	Ser–Gly–Phe–Ile–Glu–Glu–Asp–Glu–Leu–Lys	1165.55
Gln–Asn–Glu–Glu–Glu–Val–Lys	857.38	Val–Glu–Asp–Glu–Phe–Pro–Asp–Leu–Ser–Lys	1177.55
Leu–Glu–His–Glu–Glu–Ser–Lys	870.46	ln–Gly–Val–Gln–Asp–Glu–Asn–Gly–Glu–Ser–His	1182.45
Ala–Ala–Glu–Asp–Leu–Lys–Glu–Gln	902.44	Gly–Trp–Leu–Asp–Lys–Asn–Lys–Asp–Pro–Leu	1184.62
Thr–Glu–Asn–Gly–Glu–Phe–Gly–Arg	909.39	Asn–Gly–Glu–Gln–Asp–Glu–Gly–Val–Ser–His–Tyr	1234.48
Gln–Ser–Glu–Glu–Ala–Glu–Glu–Gln	931.34	Glu–Ala–Pro–Leu–Ala–Cys–Asn–Gly–Arg–Asn–Pro–Lys	1286.60
Ala–Gly–Asp–Ser–Gly–Asp–Asp–Gly–Ala–Ile–Gly	933.37	Met–Glu–Gly–Asp–Leu–Asn–Glu–Met–Glu–Ile–Gln	1308.52
Leu–Pro–Asp–Gly–Gly–Val–Ile–Leu–Gln	943.42	Asp–Leu–Glu–Ser–Glu–Val–Asp–Asn–Glu–Gln–Arg	1333.57
Asp–Lys–Gly–Asn–Gly–Glu–Thr–Val–Met	950.40	Ala–Glu–Lys–Asp–Glu–Glu–Met–Glu–Gln–Ile–Lys	1348.62
Thr–Glu–Glu–Leu–Glu–Glu–Ser–Lys	963.44	Ser–Ile–Asp–Asp–Lys–Glu–Glu–Leu–Asp–Ala–Thr–Asp	1349.58
Glu–Gln–Ile–Asp–Asn–Leu–Gln–Arg	1014.51	Ala–Ser–Glu–Gly–Asp–Asp–Asn–Leu–Asn–Ala–Glu–Glu–Arg	1418.59
Asp–Glu–Glu–Met–Glu–Gln–Ile–Lys	1020.44	Gln–Met–Met–Thr–Asn–His–Lys–Pro–Glu–Leu–Ile–Glu	1453.66
Ser–Ala–Asp–Gln–Val–Glu–Asp–Phe–Lys	1037.47	Asp–Asp–Leu–Gln–Ala–Glu–Glu–Asp–Lys–Val–Asn–Thr–Leu	1488.70
